# Robust Tracking Method for Small and Weak Multiple Targets Under Dynamic Interference Based on Q-IMM-MHT

**DOI:** 10.3390/s25041058

**Published:** 2025-02-10

**Authors:** Ziqian Yang, Hongbin Nie, Yuxuan Liu, Chunjiang Bian

**Affiliations:** 1 National Space Science Center, Chinese Academy of Sciences, Beijing 100190, China; yangziqian19@mails.ucas.ac.cn (Z.Y.); niehongbin@nssc.ac.cn (H.N.); liuyuxuan231@mails.ucas.ac.cn (Y.L.); 2 University of Chinese Academy of Sciences, Beijing 100049, China

**Keywords:** multi-target tracking, point target, multiple hypothesis tracking, interactive multiple model, adaptive model switching

## Abstract

In complex environments, traditional multi-target tracking methods often encounter challenges such as strong clutter interference and interruptions in target trajectories, which can result in insufficient tracking accuracy and robustness. To address these issues, this paper presents an improved multi-target tracking algorithm, termed Q-IMM-MHT. This method integrates Multiple Hypothesis Tracking (MHT) with Interactive Multiple Model (IMM) and introduces a Q-learning-based adaptive model switching strategy to dynamically adjust model selection in response to variations in the target’s motion patterns. Furthermore, the algorithm utilizes Support Vector Machines (SVMs) for anomaly detection and trajectory recovery, thereby enhancing the accuracy of data association and the overall robustness of the system. Experimental results indicate that under high noise conditions, the Root Mean Square Error (RMSE) of position estimation decreases to 0.74 pixels, while the RMSE of velocity estimation falls to 0.04 pixels/frame. Compared to traditional methods such as the Unscented Kalman Filter (UKF), IMM, and CIMM, the RMSE is reduced by at least 10.84% and 42.86%, respectively. In scenarios characterized by target trajectory interruptions and clutter interference, the algorithm maintains an association accuracy exceeding 46.3% even after 30 frames of interruption, significantly outperforming other methods. These findings demonstrate that the Q-IMM-MHT algorithm offers substantial performance improvements in multi-target tracking tasks within complex environments, effectively enhancing both tracking accuracy and stability, with considerable application value and extensive potential for future use.

## 1. Introduction

In the task of real-time tracking of weak and small multi-targets using infrared remote sensing, achieving high-precision and stable target tracking is essential for mission success. Due to the limitations in resolution and observation distance of space-based infrared sensors, targets typically appear as point targets on the sensor’s image plane, resulting in only limited information being available about these targets. This characteristic of point targets presents numerous challenges for target detection methods in complex environments. Strong clutter can generate high false alarm rates, which may lead to the crossing of the targets’ trajectories on the sensor’s image plane during the maneuvering of multiple targets, thereby increasing the likelihood of association errors during tracking. Furthermore, the limitations of current target detection methods may result in interrupted motion trajectories, complicating the effectiveness of traditional target-tracking approaches [1,2]. Consequently, achieving stable tracking of multiple maneuvering point targets in the presence of false alarms has emerged as a significant challenge in contemporary research.

In tracking maneuvering targets, traditional methods such as Kalman filtering [3], particle filtering [4], and correlation filtering [5] demonstrate effectiveness under certain conditions. However, their adaptability is limited when addressing complex target motion states, particularly in scenarios involving trajectory crossing and interruptions, which leads to a marked decline in tracking performance.

To address this issue, Zhang Dan [6] proposed a multi-target tracking algorithm based on the IMM. This algorithm shifts its focus away from the specific correspondence between measurements and targets, instead calculating the Mahalanobis distance between observation data and predicted observations. It then derives the association probability between targets and measurements based on this distance, ultimately obtaining state estimations through a weighted summation. Fu Xiongtao [7] and colleagues further enhanced the Adaptive Transition Probability Matrix-Interactive Multiple Model (ATPM-IMM) algorithm by incorporating an adaptive control window, which modifies the transition probability matrix. This enhancement allows for adaptive switching of maneuver models in accordance with the target’s maneuvering behavior, thereby improving the matching probability of the true model. Additionally, Ji Yunbiao [8] proposed an improved adaptive IMM algorithm that utilizes model probability estimation. This enhancement increases tracking accuracy during periods of unchanged motion states and accelerates model switching speed during motion transition phases.

However, the aforementioned methods primarily address the issue of model switching lag that arises when the target motion mode changes frequently or when there is insufficient prior information, by modifying the transition matrix in the IMM framework. Despite these improvements, a decrease in tracking accuracy may still occur. To tackle the misassociation or discontinuity in target tracking caused by trajectory crossings and interruptions, Liu Jianfeng [9] proposed an m-best hypothesis N-scan MHT method focused on measurements. This method generates m-best hypotheses for each scenario and facilitates data association by producing the optimal feasible hypothesis through an N-width sliding window. Additionally, Wang Zimei [10] and colleagues introduced an online clutter estimation MHT algorithm based on Kernel Density Estimation (KDE). This algorithm utilizes the kernel density function to model the unknown clutter density function, thereby adaptively estimating the clutter intensity within the gate at any given moment, which enhances both data association accuracy and target tracking stability.

Although existing algorithms have improved the performance of maneuvering target tracking to some extent, complex scenarios [11,12] present numerous challenges, including multiple targets with diverse motion modes, frequent trajectory crossings, and various sources of noise and interference. These factors complicate the accurate and stable tracking of multiple targets. Furthermore, the sudden appearance or disappearance of targets, along with changes in sensor noise characteristics and other uncertain factors, introduces additional challenges to multi-target tracking. To address these issues and fulfill the requirements of multi-target tracking in complex environments, this paper proposes an improved algorithm framework, Q-IMM-MHT, which integrates multiple hypothesis tracking with interactive multiple models and innovates in the following aspects:

1. In response to the issue of model switching lag in traditional IMM methods, which rely on fixed Transition Probability Matrices (TPMs) when the target motion mode changes frequently, this paper introduces a Q-learning-based adaptive model switching strategy. This approach addresses the use of incorrect models for trajectory prediction, which leads to deviations in target position estimation and ultimately compromises the accuracy and stability of tracking. By assigning a Q value to each state-action pair and continuously updating these values through interactions with sampling data, the error accumulation caused by model switching lag is mitigated. This method not only resolves the problem of model switching lag inherent in traditional IMM techniques but also enhances the accuracy, stability, and real-time performance of the tracking system. Furthermore, it improves the flexibility and precision of model switching and bolsters the system’s robustness against variations in target motion modes.

2. In multi-target tracking scenarios, target trajectories may be disrupted due to sensor data loss, clutter interference, or abrupt changes in target motion modes. The crossing of trajectories between targets further complicates data association. Traditional IMM methods often struggle to recover target trajectories promptly or to accurately associate target point traces in instances of trajectory interruption and crossing, due to model switching lag and limited data association capabilities. This results in discontinuous tracking and a significant decline in accuracy. By integrating MHT with IMM, this paper leverages the multi-trajectory hypothesis management capabilities of MHT to generate multiple potential trajectory paths during interruptions and to dynamically update association hypotheses during trajectory crossings. This ensures the correct association of target point traces, reduces misassociation and loss, and effectively addresses the challenges of trajectory interruption and target crossing.

3. In response to the challenge of misassociation of point traces under conditions of high false alarms, this paper proposes a dynamic hypothesis management strategy based on SVM. By leveraging its effective binary classification capabilities and learning the distinguishing features of targets versus clutter, the SVM can accurately identify false alarm point traces that are erroneously classified under such conditions, marking them as abnormal point traces. This approach significantly mitigates the impact of the false alarm rate on the accuracy of target association and enhances the robustness of tracking, particularly in complex environments.

4. In high-dynamic scenarios, although the MHT algorithm can address trajectory interruption issues, the generation of numerous hypotheses results in a substantial increase in computational complexity, complicating the fulfillment of real-time tracking requirements. This paper introduces a dynamic hypothesis management strategy within the MHT framework, integrating the anomaly detection capabilities of SVM into the hypothesis management process. By optimizing the generation and updating of hypotheses through trajectory scoring and hypothesis filtering mechanisms, SVM assesses the confidence of hypotheses and employs a trajectory scoring system to prioritize them. This process retains high-confidence hypotheses while eliminating redundant or invalid ones. The weights of the hypotheses are dynamically adjusted based on historical consistency and observation matching, significantly reducing unnecessary calculations and thereby enhancing computational efficiency and real-time performance.

## 2. Multi-Target Image Plane Tracking Method Based on Q-IMM-MHT

### 2.1. Overall Framework

To address the challenge of stable and real-time tracking of multiple maneuvering targets in environments with significant clutter interference, this paper introduces the Q-IMM-MHT method. This approach integrates MHT with IMM to effectively manage trajectory intersections and interruptions while dynamically adjusting motion models to accommodate the complex and variable movement characteristics of targets. Specifically, MHT is responsible for hypothesis generation and trajectory updates, whereas IMM addresses target maneuverability, significantly enhancing tracking continuity and system robustness.

The integration of MHT and IMM capitalizes on the strengths of both methodologies. MHT generates and tracks multiple target hypotheses, effectively tackling issues related to trajectory intersections and discontinuities. This is particularly advantageous in high-clutter environments, where MHT can accurately manage the confidence levels of different hypotheses, thereby improving the accuracy of multi-target tracking. Conversely, IMM utilizes multiple motion models to describe changes in target movement, dynamically switching models to enhance prediction accuracy. This adaptability makes IMM well-suited for scenarios involving complex or irregular target motions, facilitating real-time adjustments and providing optimal trajectory estimates.

The workflow of the framework is outlined as follows: First, the sensor acquires target observation data. Next, MHT generates multiple hypothesis trajectories based on initial position estimates, addressing trajectory intersections and interruptions. Subsequently, IMM dynamically adjusts the motion models based on the hypothesis trajectories to predict future target positions. Finally, the system outputs the final positions and trajectories of each target, ensuring precise tracking through continuous updates of hypotheses and models.

**Figure 1:**
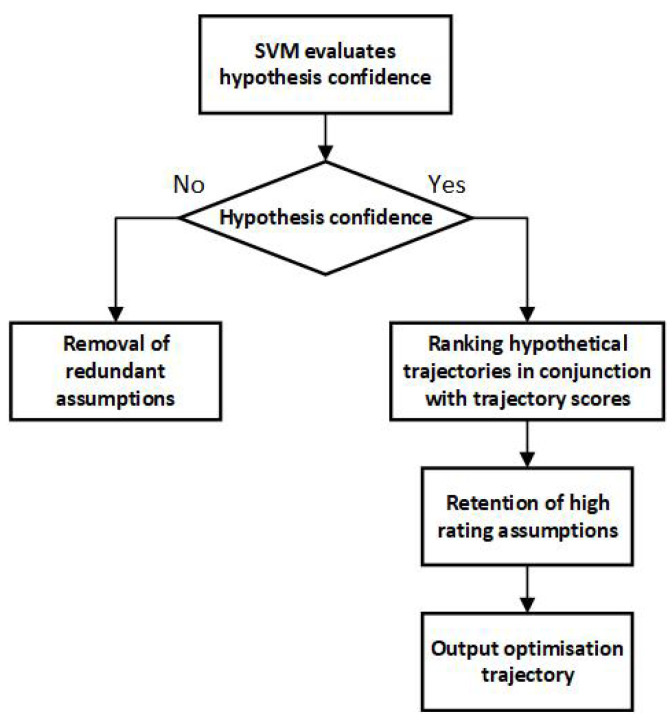
Algorithm framework of MHT with dynamic hypothesis management strategy.

Figure 1 illustrates the MHT framework, incorporating a dynamic hypothesis management strategy. Initially, the SVM model assesses whether an alarm threshold should be established for a given trajectory. If no alarm is deemed necessary, redundant trajectories are eliminated. Conversely, if an alarm is warranted, trajectories are ranked according to their scores, with high-scoring trajectories selected for the output of optimized trajectory data.

**Figure 2:**
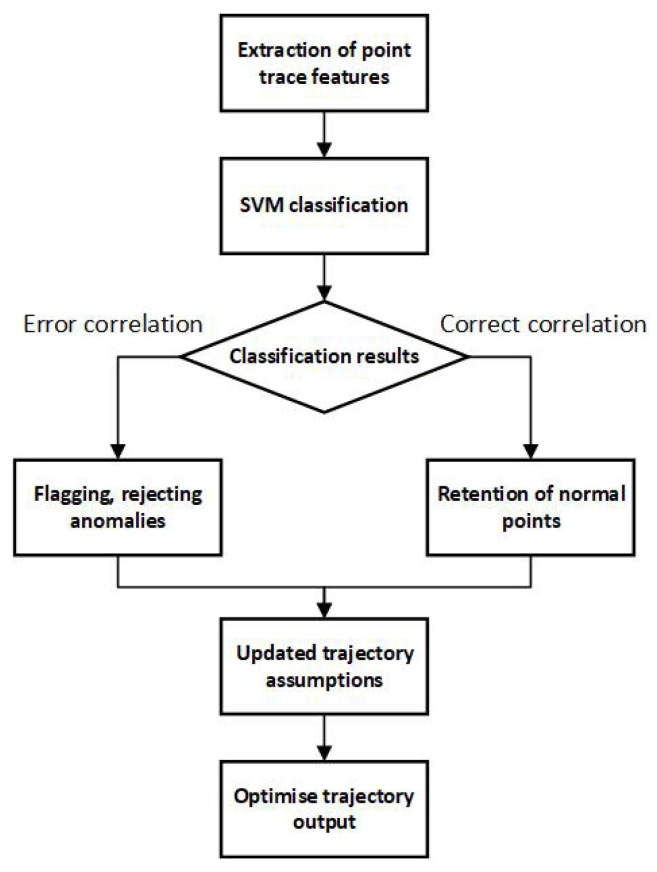
Trajectory optimization process based on SVM evaluation.

Figure 2 depicts the trajectory optimization process based on SVM evaluation. The SVM model first determines whether an alarm threshold should be set for a trajectory. If an alarm is not required, redundant trajectories are discarded. If an alarm is necessary, trajectories are sorted, and high-scoring ones are chosen to generate optimized data. This method effectively cleans and optimizes trajectories, ensuring the retention of critical information while enhancing data quality and processing efficiency.

**Figure 3:**
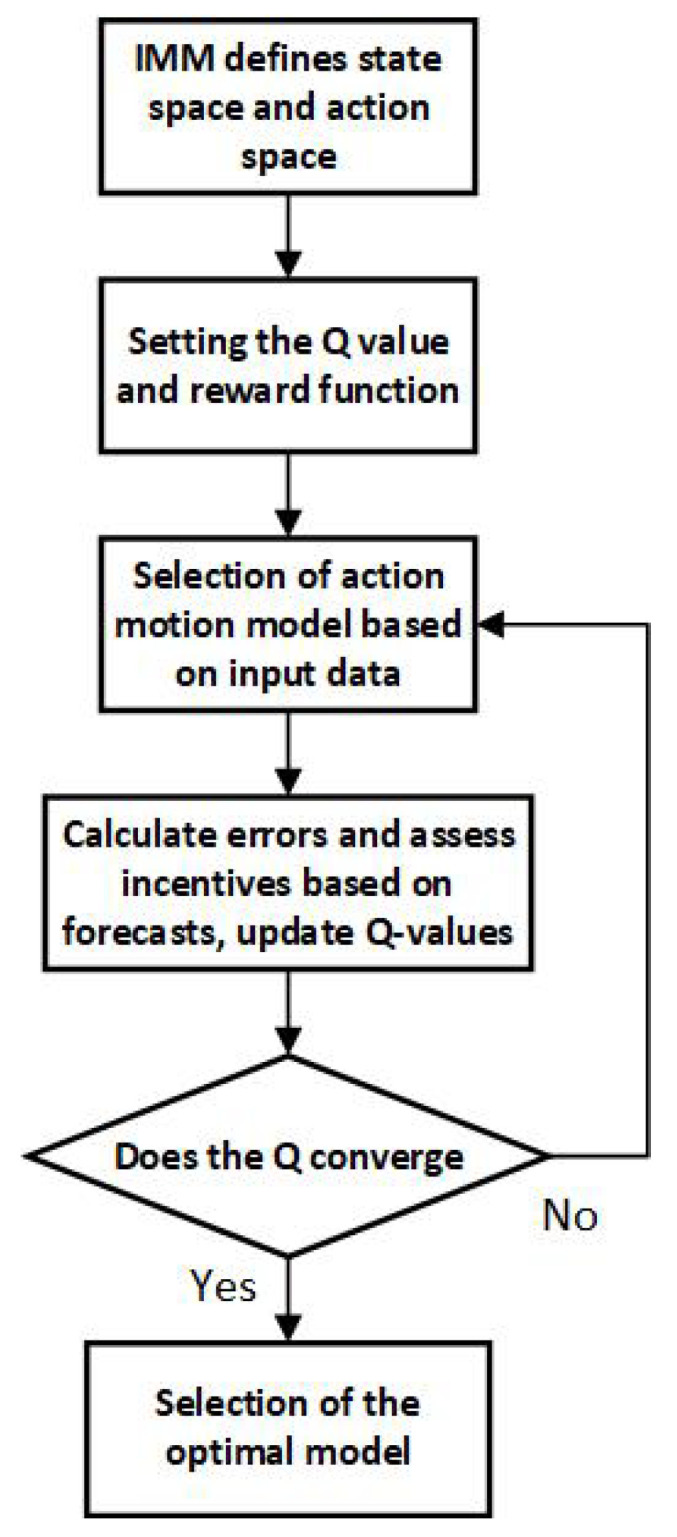
Q-IMM-MHT algorithm framework.

Figure 3 presents the dynamic model selection optimization process based on the IMM approach. This process begins by defining the state space and action space, followed by the establishment of initial Q-values and a reward function. Subsequently, actions (motion models) are selected based on input data, and prediction errors are computed to evaluate the corresponding rewards. Q-values are dynamically updated through interaction, and the process iteratively checks for Q-value convergence, ultimately selecting the optimal motion model. This reinforcement learning-based optimization of model selection enhances both prediction accuracy and adaptability.

### 2.2. Interactive Multiple Models

#### 2.2.1. Principles

The IMM [13,14] approach is a filtering method that employs various motion models to improve the accuracy of target tracking in complex motion scenarios. IMM dynamically transitions between different motion models, such as constant velocity and constant acceleration, in response to fluctuations and uncertainties in target movement. It automatically adjusts the switching weights among the models based on observational data, thereby facilitating precise predictions and state estimations.

**Figure 4:**
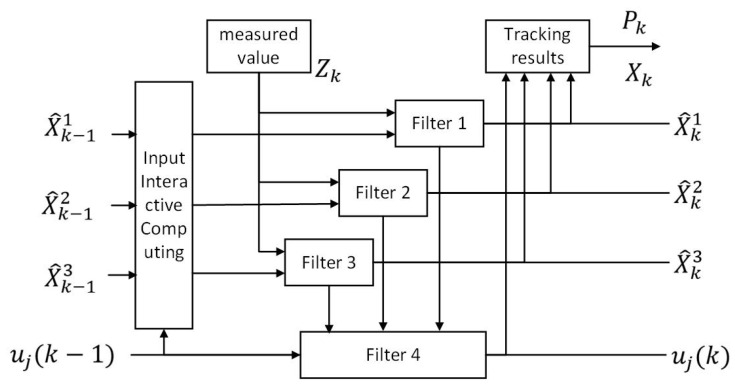
Principle of IMM.

Figure 4 illustrates the principle of the IMM framework [15]. Within this framework, multiple models simultaneously estimate the target state and dynamically update the switching probabilities between these models using a TPM [16]. When new observational data are received, IMM recalibrates the model weights based on posterior and transition probabilities, determining the most suitable model for predictions and updates. This adaptive mechanism effectively addresses dynamic changes in target movement, resulting in enhanced tracking precision.

#### 2.2.2. Dynamic Model Switching and Prediction Strategy

In dynamic target tracking, the motion patterns of targets often change. Traditional IMM methods adapt to these changes by concurrently computing multiple motion models and blending their predictions through weighted averaging. However, this approach depends on predefined model switching rules, which lack the capability for adaptive optimization. To address this limitation, this paper proposes a dynamic model switching and prediction strategy that integrates reinforcement learning, specifically employing Q-learning to intelligently optimize model switching within the IMM framework.

Q-learning [17] is a value-based reinforcement learning algorithm that guides model selection by learning the expected returns of each state–action pair. In the proposed method, the system not only predicts the target’s position and velocity but also dynamically adjusts the model selection strategy based on model adaptability. With the introduction of Q-learning, the model selection process is transformed into a decision-making procedure, defined as follows: State Space:

(1) $$s_{t}=\left\{x_{t},v_{t},{\hat{\mu }}_{\mathrm{CV}}\left(t\right),{\hat{\mu }}_{\mathrm{CA}}\left(t\right),{\hat{\mu }}_{\mathrm{CT}}\left(t\right)\right\}$$

where $x_{t}$ is the target position, $v_{t}$ is the target velocity, and ${\hat{\mu }}_{i}\left(t\right)$ represents the IMM-estimated weight for each model, reflecting model adaptability. Action Space:

(2) A={CV,CA,CT}

Each action corresponds to selecting a specific motion model for prediction and update: Constant Velocity (CV), Constant Acceleration (CA), and Constant Turn Rate (CT). In the context of tracking within the sensor’s image plane, the target’s motion remains relatively simple in two dimensions, despite potential maneuvers, due to the long distances involved and projection effects. Consequently, the traditional CV, CA, and CT models effectively characterize the target’s motion state. The reward function assesses the effectiveness of each model selection. In dynamic target tracking, the primary objective is to minimize tracking errors; thus, the reward function is formulated as the negative value of the prediction error.

(3) $$r_{t}=-\left(|\right|{\hat{x}}_{t}-x_{t}{\left|\right|}^{2}+\left|\right|{\hat{v}}_{t}-v_{t}{\left|\right|}^{2})$$

where ${\hat{x}}_{t}$ and ${\hat{v}}_{t}$ are the predicted position and velocity obtained by the selected model, and $x_{t}$ and $v_{t}$ are the actual observed values. This reward function encourages the model selection strategy to minimize errors, thereby enhancing tracking accuracy.

The objective of Q-learning is to select action $a_{t}$ (motion model) at each state $s_{t}$ to maximize the cumulative reward. The core update formula is:

(4) $$Q\left(s_{t},a_{t}\right)\leftarrow Q\left(s_{t},a_{t}\right)+\alpha \left[r_{t}+\gamma *max_{a^{\prime }}Q\left(s_{t+1},a^{\prime }\right)-Q\left(s_{t},a_{t}\right)\right]$$

where α is the learning rate and γ is the discount factor.

In the IMM framework, the state AAA represents the target’s current motion mode, while the action BBB corresponds to the chosen motion model (e.g., constant velocity, constant acceleration, etc.). The Q-learning algorithm updates the Q-values based on the target’s motion state, observational data, and prior experiences, thereby selecting the optimal motion model according to the updated Q-values.

This approach allows Q-learning to automatically learn the most appropriate model selection strategy for the current target motion state, effectively addressing the limitations of the traditional IMM method, which relies on a fixed TPM that cannot adapt to dynamic environmental changes. Unlike traditional methods, Q-learning does not depend on prior knowledge; instead, it optimizes the model switching strategy through the accumulation of experience and interactive learning. Over time, Q-learning dynamically adjusts the model switching probabilities, enabling the IMM framework to rapidly select the optimal model as the target’s motion mode evolves, thereby enhancing the flexibility of model switching and the overall robustness of the system.

#### 2.2.3. Q-Learning Implementation for Dynamic Model Switching

The integration of Q-learning with the IMM framework seeks to mitigate the model switching delay associated with the traditional IMM methods due to the TPM. Q-learning dynamically refines the model selection strategy via a reinforcement learning mechanism, thereby enhancing adaptability to variations in target motion patterns. The specific implementation steps are as follows:

1. The state space, denoted as ABC, encompasses the target’s current position ($\chi_{t}$), velocity ($v_{t}$), and the weights of each model in the Interacting Multiple Model (IMM), specifically ${\hat{\mu }}_{\mathrm{CV}}\left(t\right),{\hat{\mu }}_{\mathrm{CA}}\left(t\right),{\hat{\mu }}_{\mathrm{CT}}\left(t\right)$. These weights indicate the degree to which each model adapts to the target’s current motion state. The action space, represented as A = CV, CA, CT, corresponds to three motion models: constant velocity, constant acceleration, and constant turn.

2. The reward function $r_{t}$ is determined by the model’s prediction error, which is calculated as the negative squared Euclidean distance between the predicted position ${\hat{x}}_{t}$ and velocity ${\hat{v}}_{t}$, and the actual observed values $\chi_{t}$ and $v_{t}$. This design seeks to minimize prediction errors and ensures that the model selection strategy rapidly adapts to changes in the target’s motion pattern.

3. Q-value update and model selection: Q-learning updates the Q-values using the Bellman equation, and these updated Q-values dynamically adjust the IMM model weights $\mu_{i}\left(t+1\right)$ through the Softmax function, replacing the traditional fixed TPM. The formula is:

$$\mu_{i}\left(t+1\right)=\frac{\exp(Q\left(s_{t},a_{i}\right))}{\sum_{j}\exp\left(Q\left(s_{t},a_{j}\right)\right)}$$

This mechanism facilitates a more accurate model transition in accordance with the actual motion patterns of the target. For example, when the target experiences a sudden acceleration, Q-learning rapidly increases the weight of the CA model, thereby minimizing prediction delays.

4. Collaboration mechanism with MHT: Following the generation of multiple hypothesis trajectories by MHT, Q-IMM-MHT independently executes the Q-learning module for each hypothesis, assessing the adaptability of each model to the proposed trajectory. The model that yields the highest Q-value is selected as the optimal prediction, and target state estimation is produced through weighted fusion, thereby ensuring continuity in tracking during complex motion scenarios.

### 2.3. Multi-Hypothesis Tracking

MHT [18] is widely utilized in the field of multi-target tracking, particularly for addressing complex data association challenges in high-clutter environments. By maintaining multiple trajectory hypotheses simultaneously, MHT enhances tracking performance in uncertain and dynamic settings, systematically exploring various data association possibilities. Compared to traditional single-hypothesis tracking methods, MHT more effectively manages target maneuver changes, trajectory intersections, and association errors induced by strong clutter. Additionally, it resolves tracking interruptions resulting from target detection issues, significantly improving the accuracy and robustness of multi-target tracking.

#### 2.3.1. Principles

The fundamental workflow of MHT consists of four steps: hypothesis generation, data association, hypothesis management, and trajectory update. At each time step, the system generates new trajectory hypotheses based on the current observation data and prior trajectory hypotheses [19]. Each hypothesis represents a potential motion path of a target, encompassing various data association scenarios. This methodology enables the system to maintain multiple trajectory hypotheses at any given moment, thereby avoiding misassociations that may arise from sudden trajectory changes or intersections.

During the data association phase, MHT determines the matching relationships between observation points and trajectory hypotheses by calculating the association probabilities between them. In contrast to Joint Probabilistic Data Association (JPDA) [20], MHT evaluates the joint probabilities between observation points and multiple targets, selecting the most probable association paths to mitigate the risk of target loss and misassociations.

1. Despite MHT’s remarkable performance in enhancing the stability of multi-target tracking, particularly in cluttered environments, it still encounters several challenges: 1. High Computational Complexity: The necessity of maintaining multiple trajectory hypotheses and simultaneously computing association probabilities significantly increases the computational burden as the number of targets and the volume of data grows.

2. Sensitivity to Clutter: When target trajectories change abruptly or new targets emerge, errors can occur during the hypothesis management and updating processes. These errors may lead to an unnecessary increase in hypotheses, consequently affecting tracking accuracy and efficiency.

To address these challenges, this paper proposes the integration of SVM with MHT to enhance performance. SVM, recognized as a robust classification tool, effectively manages nonlinear features and exhibits strong generalization capabilities. By incorporating SVM into multi-hypothesis tracking, the system can learn and classify the relationships between input measurements and trajectory hypotheses, thereby improving data association accuracy and reducing the likelihood of misassociations. This integration significantly enhances overall tracking performance in high-clutter and multi-target environments.

#### 2.3.2. Anomaly Detection and Recovery Mechanism

In high-clutter environments, significant clutter can lead to false detections and incorrect tracking, which adversely affects overall tracking performance. To ensure that the system can swiftly recover accurate tracking after trajectory interruptions, this paper introduces an anomaly detection and recovery mechanism within the MHT framework. This mechanism consists of four components: an anomaly detection module, misassociation handling, trajectory recovery, and dynamic hypothesis expansion.

1. Anomaly Detection Mechanism.

The anomaly detection module is designed to identify abnormal measurements caused by strong clutter in real-time, thereby reducing misassociations and incorrect tracking. This study employs SVM as the primary method for anomaly detection. Initially, features such as position, velocity, and acceleration are extracted from the observation data and utilized as inputs to the SVM to differentiate between normal and abnormal measurements. During the training phase, a binary classification SVM model is developed using a labeled historical dataset that includes both normal and abnormal measurements. The SVM effectively separates normal and abnormal measurements by employing an optimal hyperplane, with the decision function defined as:

(5) $$f\left(x\right)=w^{T}x+b$$

where *x* is the input feature vector, *w* is the weight vector, and *b* is the bias term. When f(x)≥0, the measurement is classified as normal; otherwise, it is deemed abnormal. The SVM optimizes the objective function to find the optimal hyperplane:

(6) $$*min_{w,b}\frac{1}{2}{∥w∥}^{2}+C\sum_{i=1}^{N}\xi_{i}$$

subject to:

(7) $$y_{i}\left(w^{T}x_{i}+b\right)\geq 1-\xi_{i},\xi_{i}\geq 0,\forall i=1,2,\ldots ,N$$

where $y_{i}$ represents the class label of sample *i*, while $xi_{i}$ denotes the slack variables. The parameter *C*, which is greater than zero, serves as the penalty parameter that regulates the trade-off between maximizing the margin and minimizing classification errors. By addressing this optimization problem, the anomaly detection module can effectively identify abnormal measurements, thereby enhancing both detection accuracy and robustness.

2. Misassociation Handling Mechanism.

Upon detecting abnormal measurements, it is crucial to address misassociations to maintain tracking accuracy. The process of misassociation handling encompasses hypothesis verification and trajectory scoring. Initially, trajectory hypotheses within the MHT framework undergo consistency checks to discard those that do not align with the established motion models. Specifically, the state transition of trajectory hypothesis $H_{t}$ is represented as:

(8) $$x_{t|t-1}^{\left(i\right)}=Fx_{t-1}^{\left(i\right)}+\gamma w_{t-1}^{\left(i\right)}$$

where *F* is the state transition matrix, γ is the process noise coefficient, and $w_{t-1}$ represents process noise. The observation residual covariance matrix $S_{t}$ is given by:

(9) $$S_{t}=HP_{t|t-1}H^{T}+R$$

where $P_{t|t-1}$ is the prior state estimate covariance matrix, and *R* is the observation noise covariance matrix. Using the Chi-square test:

(10) $$y_{t}^{(i)T}S_{t}^{-1}y_{t}^{\left(i\right)}\leq \chi_{\alpha }^{2}\left(n\right)$$

the consistency of hypothesis $H_{i}$ is evaluated, where $\chi_{\alpha }^{2}\left(n\right)$ is the critical value of the Chi-square distribution with *n* degrees of freedom.

Subsequently, a trajectory scoring function evaluates the credibility of each trajectory hypothesis:

(11) $$S\left(H_{i}\right)=\lambda_{1}\cdot C_{\mathrm{history}}\left(H_{i}\right)+\lambda_{2}\cdot C_{\mathrm{current}}\left(H_{i}\right)$$

This scoring prioritizes high-scoring trajectories, thereby reducing the impact of misassociations.

3. Trajectory Recovery Mechanism After a target trajectory is interrupted, the system establishes a recovery window to predict potential motion paths based on historical observations and motion model predictions. By integrating IMM motion models, several predicted trajectory paths are generated.

(12) $${\hat{x}}_{t|t-1}=F{\hat{x}}_{t-1|t-1}+\gamma w_{t}$$

Future positions and velocities are forecasted, and subsequent observation measurements are re-associated to accurately restore the correct trajectory.

4. Dynamic Hypothesis Expansion Mechanism MHT addresses uncertainties in target motion and the loss of observation data by generating additional trajectory hypotheses. However, prolonged interruptions can result in an exponential increase in the number of hypotheses, thereby escalating computational complexity. To mitigate this issue, a probability-based dynamic hypothesis expansion strategy is proposed, which generates hypotheses only for those with high association probabilities or based on prior knowledge.

(13) $$Z_{t}^{\left(j\right)}=H{\hat{x}}_{t|t-1}^{\left(j\right)}+v_{t}^{\left(j\right)}$$

To re-associate predicted trajectories with observation data, the system calculates the association probability between the observation $Z_{t}^{\left(j\right)}$ and the trajectory hypothesis H:

(14) $$P\left(z_{t}|H_{j}\right)=\frac{1}{\sqrt{{\left(2\pi \right)}^{n}\left|S_{t}^{\left(j\right)}\right|}}e^{-\frac{1}{2}{\left(z_{t}-H{\hat{x}}_{t|t-1}^{\left(j\right)}\right)}^{T}{\left(S_{t}^{\left(j\right)}\right)}^{-1}\left(z_{t}-H{\hat{x}}_{t|t-1}^{\left(j\right)}\right)}$$

The system dynamically updates the hypothesis weights based on the association probabilities:

(15) $$\varepsilon_{t}^{\left(j\right)}=\frac{P(z_{t}\left|H_{j}\right)}{\sum_{k}P\left(z_{t}|H_{k}\right)}$$

During hypothesis expansion, new hypotheses $H_{j}$ are generated based on different prediction models and observation data, introducing a matching score function for new hypothesis paths and motion models:

(16) $$S(H_{j})=\lambda_{1}\cdot C_{\mathrm{history}}(H_{j})+\lambda_{2}\cdot C_{\mathrm{current}}(H_{j})+\lambda_{3}\cdot C_{\mathrm{model}}(H_{j})$$

High-scoring hypotheses are retained to manage the size of the hypothesis space.

By incorporating an anomaly detection and recovery mechanism, the Q-IMM-MHT algorithm effectively identifies and addresses abnormal measurements in high-clutter environments, promptly recovering interrupted trajectories and ensuring the continuity and robustness of multi-target tracking. The integration of a probability-based dynamic hypothesis expansion strategy enables the system to maintain high tracking performance while significantly reducing computational complexity, thereby enhancing the algorithm’s efficiency and practicality.

#### 2.3.3. Mechanisms of SVM Implementation in MHTs

SVM are primarily utilized within the MHT framework for anomaly detection and trajectory recovery, specifically targeting the challenge of misassociation between target tracks and clutter tracks in high-clutter environments. The implementation mechanism consists of the following steps:

1. Feature extraction and preprocessing involve extracting the target’s position (x,y), velocity $\left(v_{x},v_{y}\right)$, and acceleration $\left(a_{x},a_{y}\right)$ from the observation data as raw features. To eliminate dimensional discrepancies, Z-score normalization is applied to standardize these features, using the following formula:

$$z=\frac{x-\mu }{\sigma }$$

where μ and σ represent the mean and standard deviation of the respective feature. This approach reduces computational complexity and enhances the robustness of classification.

2. In the process of model training and optimization, a labeled historical dataset comprising both normal and clutter tracks is utilized. This dataset is divided into training and testing sets in a 7:3 ratio. The hyperparameters of the SVM are optimized using a grid search. The Radial Basis Function (RBF) is selected as the kernel function, represented by the expression:

$$K\left(x_{i},x_{j}\right)=\exp(-\gamma ∥x_{i}-x_{j}{∥}^{2})$$

where γ=0.1 controls the kernel bandwidth. Additionally, the penalty factor C is chosen from the set 0.1, 1, 10 to balance the classification margin against the weight of misclassified samples.

3. Real-time detection and dynamic updating: Upon receiving a new observation point in each frame, standardized features are extracted and input into the SVM classifier. If the classification result indicates an abnormal (f(x)<0), it is designated as a clutter track, which triggers the trajectory recovery module to generate alternative hypotheses. Normal tracks are directly involved in the MHT association computation. To accommodate dynamic environmental changes, the SVM model is updated every 100 frames through incremental learning; the historical data window is retained, and the model parameters are retrained to ensure that the classifier adapts to new types of clutter.

4. Collaboration mechanism with Q-learning: The anomaly detection results from the SVM operate in conjunction with Q-learning’s model selection strategy. When the SVM identifies a track as clutter, Q-learning disregards the prediction error for that track to avoid interference with model weight updates. Conversely, the prediction error from normal tracks is utilized to optimize the Q-value, thereby enhancing the accuracy of model selection.

## 3. Experiments and Results

### 3.1. Experimental Setup

To validate the effectiveness of the Q-IMM-MHT method, this study compares it with the UKF, IMM, and CIMM algorithms, focusing particularly on the evaluation of association performance during target maneuvering and trajectory intersections within interruption intervals. The experimental scenarios simulate target trajectory interruptions and intersections amid strong false alarm interference. The sensor sampling time is set to 0.25 s, with interruption durations being integer multiples of this sampling time. A total of 5000 sets of simulated data are utilized, each containing five target trajectories. The sensor introduces a measurement error of three pixels along with Gaussian noise. By varying the noise coefficients, the robustness of the methods under inaccurate model descriptions is assessed. Evaluation metrics include Root Mean Square Error (RMSE) for position and velocity estimation, model probability error, and position and velocity errors under different noise coefficients. Additionally, the study evaluates tracking capabilities under scenarios of target mis-detection and trajectory intersection.

### 3.2. Experimental Results

1. Analysis of Position and Velocity Estimation Errors.

Accurate estimation of target position and velocity is crucial for multi-target tracking in complex interference environments. This study analyzes the performance of the Q-IMM-MHT algorithm in position and velocity estimation, comparing it with the UKF, IMM, and CIMM methods.

**Figure 5:**
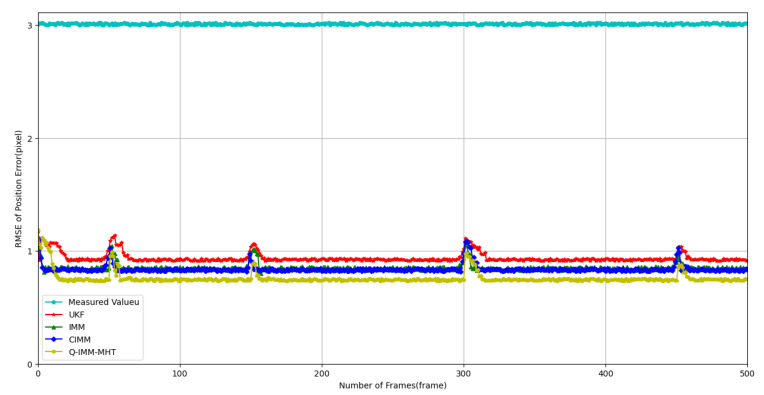
Position estimation Root Mean Square Error (RMSE) for different methods.

**Figure 6:**
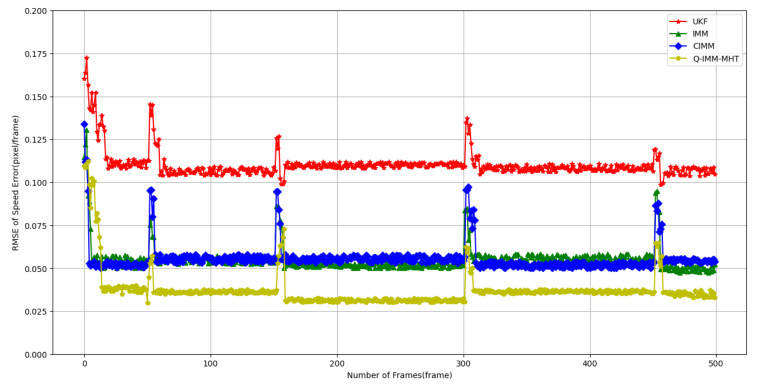
Velocity estimation Root Mean Square Error (RMSE) for different methods.

Figure 5 and Figure 6 display the RMSE for position and velocity estimation across different methods. The experimental results indicate that:

In Figure 5, the position estimation RMSE is illustrated, with the light blue line representing the measurement error value, which is approximately 3 pixels. The UKF exhibits RMSE values ranging from 0.92 to 1.10 pixels, characterized by relatively large and highly fluctuating errors. The IMM algorithm reduces the RMSE to a range of 0.84 to 1.03 pixels, while the CIMM further decreases it to between 0.83 and 1.01 pixels. The Q-IMM-MHT algorithm maintains a stabilized RMSE of 0.74 pixels, demonstrating superior stability and accuracy.

Figure 6 presents the velocity estimation RMSE, where the UKF’s velocity RMSE ranges from 0.10 to 0.15 pixels/frame, again exhibiting relatively large and fluctuating errors. The IMM reduces this range to between 0.07 and 0.10 pixels/frame, and the CIMM shows slightly better performance. The Q-IMM-MHT algorithm maintains an RMSE below 0.04 pixels/frame, showcasing exceptional performance in velocity estimation.

**Figure 7:**
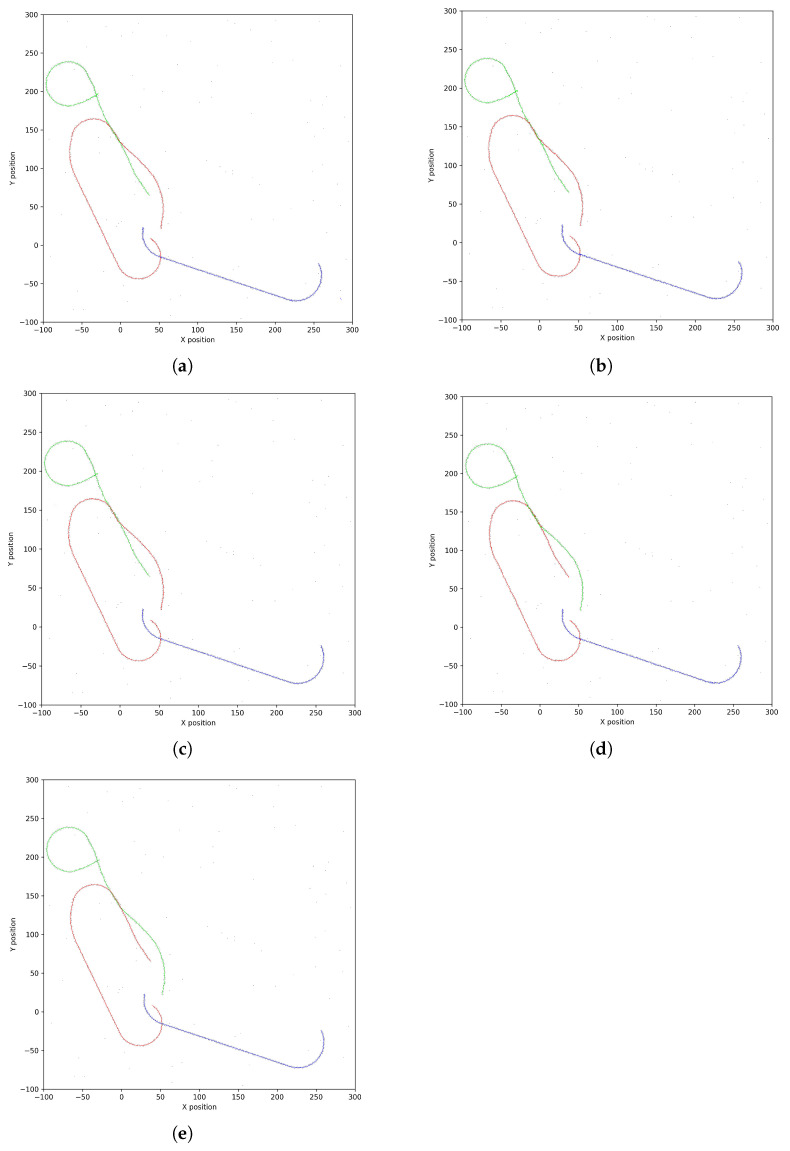
Target trajectory diagrams: (**a**) UKF. (**b**) IMM. (**c**) CIMM. (**d**) Q-IMM—MHT. (**e**) Real trajectory. (Different colors represent different trajectories).

Figure 7 illustrates the target trajectory association results for different methods. The UKF encounters association errors during trajectory intersections, leading to deviations in the trajectory. The IMM also experiences misassociations during small-angle intersections. Although the CIMM converges faster under intersection conditions, its association accuracy remains insufficient during small-angle intersections. In contrast, the Q-IMM-MHT algorithm, by combining multi-hypothesis tracking and reinforcement learning, exhibits enhanced tracking performance and robustness in small-angle intersection scenarios.

2. Analysis of Model Probability Errors.

**Figure 8:**
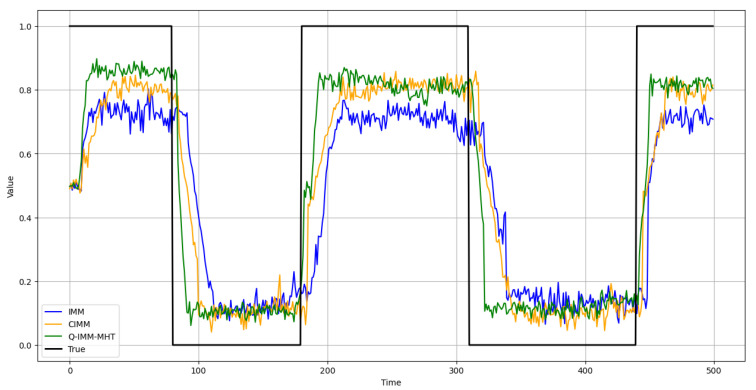
CV model probability estimates.

**Figure 9:**
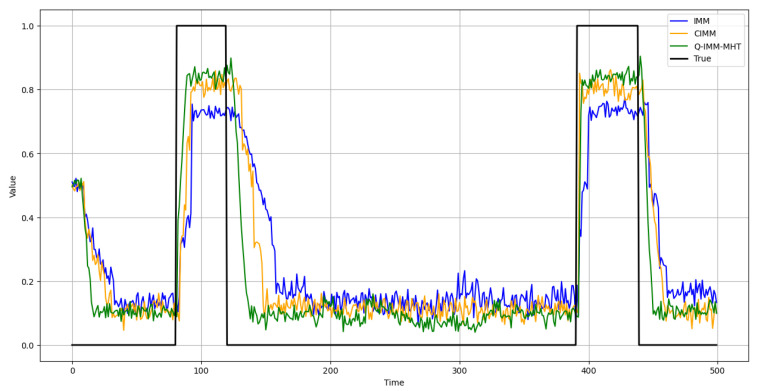
CA model probability estimates.

**Figure 10:**
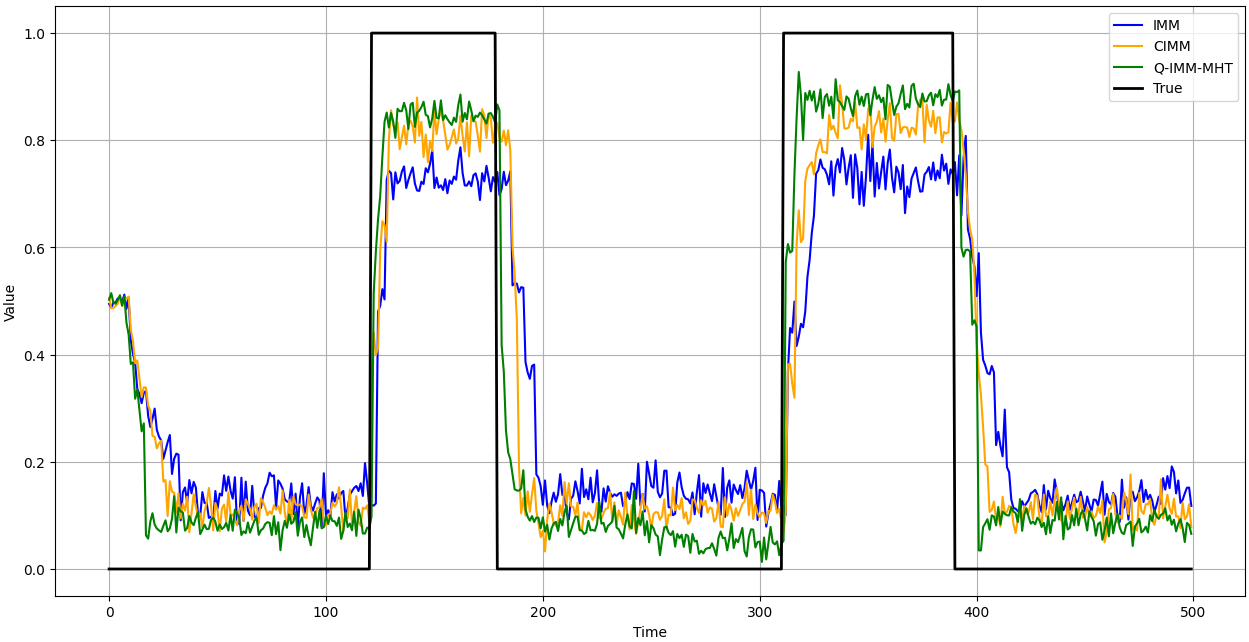
CT model probability estimates.

This study evaluates the accuracy of model probability estimation. Figure 8, Figure 9 and Figure 10 illustrate the probability estimates for the CV, CA, and CT models, respectively. In the experimental scenarios, targets execute various motion models across different time segments.

The results indicate that the IMM’s model probability curves exhibit significant deviations from the true model probabilities, particularly when the adaptation to changes in target motion patterns is not prompt. While the CIMM algorithm outperforms the IMM in model probability estimation, it still demonstrates discrepancies during specific motion model transitions. Conversely, the Q-IMM-MHT algorithm’s model probability estimates closely align with the true model probabilities, accurately identifying the actual motion states of the targets and swiftly adjusting model probabilities during changes in motion patterns, thereby maintaining tracking accuracy.

Although refined modeling with centralized models can significantly enhance the performance of the IMM algorithm in systems where target motion patterns are relatively stable and well-defined, the challenge of precise modeling increases markedly in systems characterized by inaccurate models or mismatched observations. To evaluate the robustness of the Q-IMM-MHT algorithm against noise under inaccurate model descriptions, experiments were conducted with different process noise coefficients.

(17) $$\gamma \in \{10^{-2},10^{-1.75},10^{-1.5},10^{-1.25},10^{-1},10^{-0.75},10^{-0.5},10^{-0.25},10^{0}\}$$

**Figure 11:**
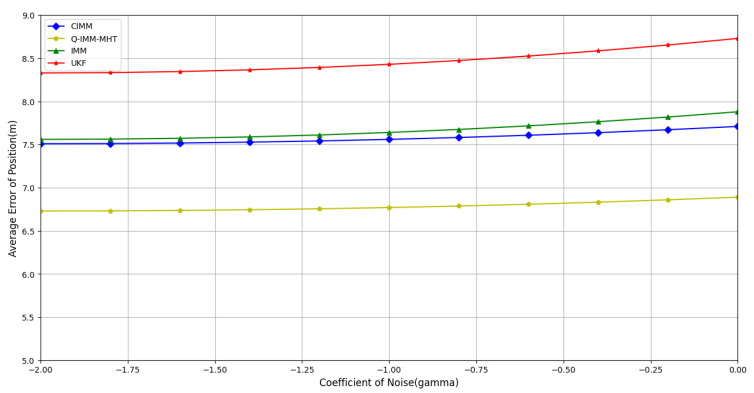
Average position estimation error under different noise coefficients.

**Figure 12:**
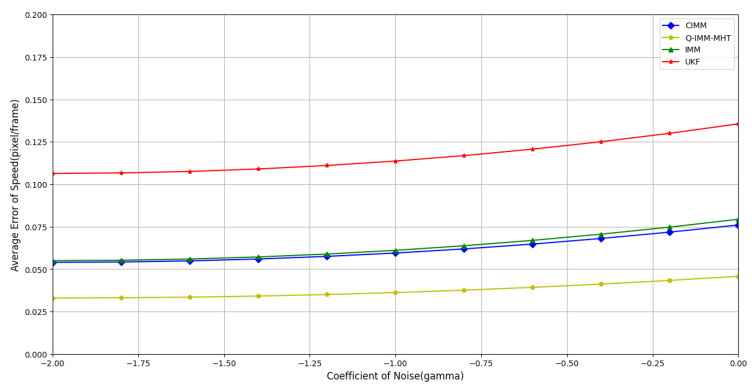
Average velocity estimation error under different noise coefficients.

**Figure 13:**
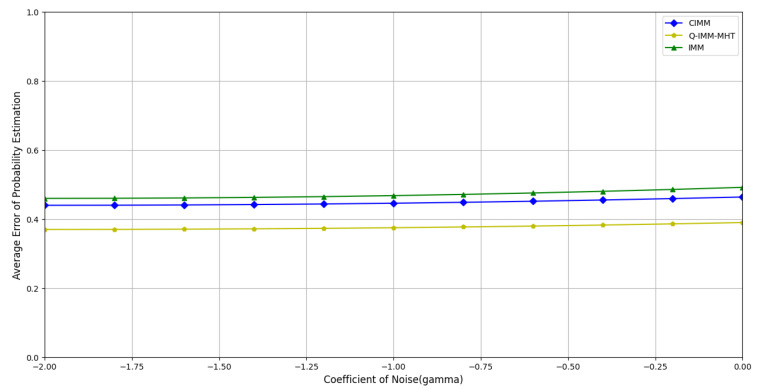
Average model probability estimation error under different noise coefficients.

A total of 500 Monte Carlo simulations were performed. Figure 11, Figure 12 and Figure 13 display the average errors in position estimation, velocity estimation, and model probability estimation under varying noise coefficients, respectively.

Figure 11 and Figure 12 clearly illustrate that as γ increases, the average estimation errors for both position and velocity rise across all algorithms. Notably, the Q-IMM-MHT algorithm consistently demonstrates significantly lower errors compared to the other methods, highlighting its strong robustness.

Figure 13 further illustrates the model probability estimation errors under various noise conditions. The Q-IMM-MHT method effectively evaluates the target’s motion models, thereby reducing the frequency of model switching errors. In contrast, the IMM and CIMM algorithms exhibit unstable model switching, leading to substantial increases in probability estimation errors when faced with inaccurate model descriptions.

3. Impact of Trajectory Intersection and Interruption on Tracking.

To evaluate the performance of the proposed algorithm, a simulated environment was established featuring five targets. These targets maintain a constant velocity and follow a straight-line trajectory both before and after interruption events. During the interruption intervals, each target experiences distinct motion states as outlined below:

Target 1: Maintains constant velocity (CV) throughout the interruption interval.

Target 2: Transitions to constant acceleration (CA) with an acceleration range of 0 to 5 m/s² during the interruption interval.

Target 3: Transitions to constant acceleration (CA) with an acceleration range of 5 to 10 m/s² during the interruption interval.

Target 4: Switches to constant turn (CT) with an angular velocity ranging from 0 to π/50 rad/frame during the interruption interval.

Target 5: Executes a constant turn flight with an angular velocity between π/50 and π/35 rad/frame.

The motion models of the targets switch randomly across various time segments, allowing for an assessment of the algorithm’s adaptability and accuracy in dynamic environments. Different lengths of interruption intervals, denoted as *K*, were established, and 500 Monte Carlo simulations were performed utilizing the UKF, IMM, CIMM, and Q-IMM-MHT algorithms to associate interrupted trajectories. As *K* increases, the number of correct associations for each algorithm is presented in Table 1.

Table 1 illustrates that the UKF maintains correct association counts ranging from approximately 66 to 122 times at K = 6, but fails completely at K = 18. In contrast, the IMM and the CIMM sustain correct association counts between 150 and 400 times over short to medium intervals (K = 6 to K = 12), although their accuracy considerably declines at K ≥ 18. The Q-IMM-MHT algorithm, however, demonstrates consistent excellence across all interruption intervals, maintaining over 200 correct associations even at K = 30.

This performance significantly surpasses that of the other algorithms, highlighting its strong adaptability and robustness in varying environmental conditions. Furthermore, the proposed algorithm exhibits remarkable adaptability to targets undergoing maneuvers during interruption intervals. Under the various target maneuver intensities and interruption interval conditions detailed in Table 1, the proposed algorithm consistently achieves high correct association counts.

Table 2 further compares the performance of different algorithms regarding global association accuracy ($R_{ga}$), average association accuracy ($R_{ta}$), average association error rate ($R_{fa}$), and average missed association rate ($R_{na}$). The Q-IMM-MHT algorithm significantly outperforms the UKF, IMM, and CIMM algorithms in both global association accuracy and average association accuracy, while also demonstrating notably lower average association error rates and average missed association rates. For example, at an interruption interval of K = 6, the global association accuracy and average association accuracy of the Q-IMM-MHT algorithm both reach 100%, which is substantially higher than those of the other algorithms. Even at K = 30, the global association accuracy of Q-IMM-MHT remains at 46.3%, significantly surpassing the performance of the UKF, IMM, and CIMM algorithms.

**Figure 14:**
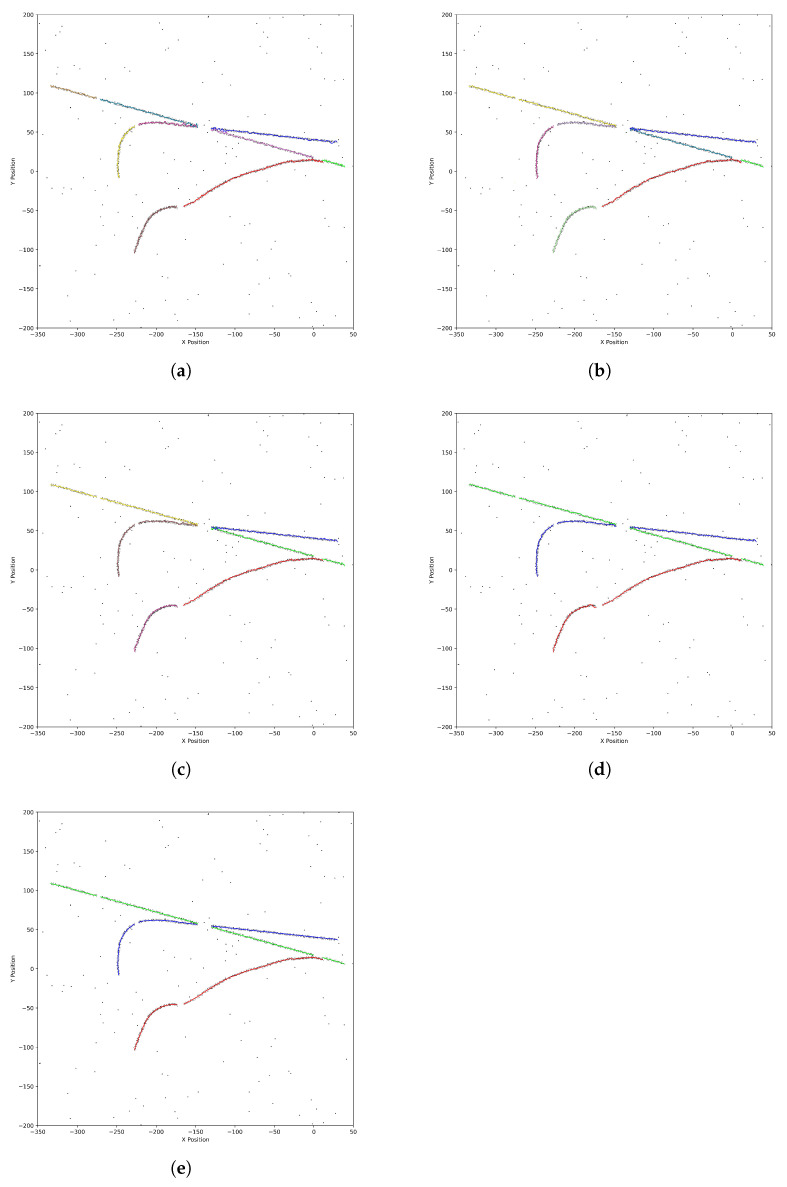
Association results under trajectory crossing and interruption: (**a**) UKF. (**b**) IMM. (**c**) CIMM. (**d**) Q-IMM—MHT. (**e**) real trajectory. (Different colors represent different trajectories).

Figure 14 illustrates the association results of various algorithms under scenarios involving trajectory crossing and interruption. The UKF exhibits a decline in association accuracy during trajectory crossings and struggles to maintain continuity following interruptions. In contrast, the IMM shows some improvement in managing trajectory crossings; however, it still encounters continuity challenges after interruptions. The CIMM demonstrates enhanced performance in addressing both trajectory crossings and interruptions. The Q-IMM-MHT algorithm stands out for its superior association accuracy, effectively minimizing misassociations and swiftly restoring tracking continuity.

4. Computational efficiency analysis with the introduction of SVM.

This study significantly enhances the real-time performance and robustness of multi-target tracking in complex environments by integrating Support Vector Machine (SVM) anomaly detection with a dynamic hypothesis management mechanism into the Multi-Hypothesis Tracking (MHT) framework. As can be known from Table 3, under the constraint of a sampling frequency of 0.25 s per frame, the introduction of the SVM method reduced the per-frame processing time from the original range of 0.3–0.36 s to 0.16–0.24 s, achieving an efficiency improvement of 34–47%. The optimized algorithm not only effectively alleviates data backlog issues caused by computational delays but also enhances the system’s real-time margin from negative values (–0.05–0.11 s per frame) to positive values (0.01–0.09 s per frame), facilitating near-real-time stable tracking in typical high-clutter scenarios (≤100 clutter points per frame). Furthermore, through the SVM-driven trajectory scoring and hypothesis pruning strategy, the system’s target capacity and clutter density tolerance were improved by 60% and 100%, respectively. This demonstrates the strong adaptability and scalability of the proposed method in dynamic environments, laying a theoretical and technical foundation for future integration with hardware acceleration or parallel optimization.

5. Sensitivity analysis of hyperparameters.

To evaluate the impact of the learning rate α on the model’s convergence speed and tracking accuracy, this paper conducted a grid search within the range of 0.01, 0.7, and 0.9, while keeping the discount factor γ fixed at 0.7 and the exploration rate ϵ fixed at 0.3. For each value of α, 500 Monte Carlo simulations were performed, and the position RMSE and the number of convergence steps—defined as the number of iterations required for the Q-values to fluctuate by less than 1%—were recorded.

**Figure 15:**
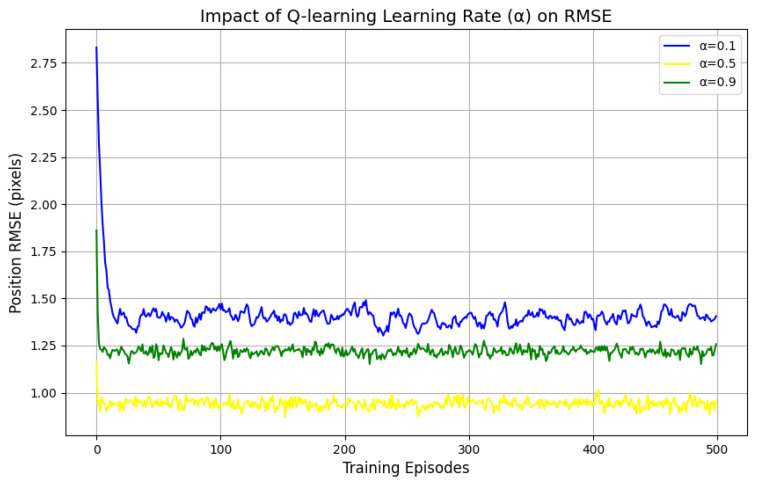
Impact of Q-learning learning Rate on RMSE.

According to Figure 15, it can be known that:

- α=0.01: xonvergence steps = 42, RMSE = 1.4 pixels, indicating slow updates leading to delayed convergence.
- α=0.7 xonvergence steps = 12, RMSE = 0.94 pixels, which balances convergence speed and stability.
- α=0.9 xonvergence steps = 10, with an RMSE fluctuation range expanding to 1.22 pixels.

For the SVM analysis, we evaluated the Radial Basis Function (RBF) kernel, Sigmoid kernel, linear kernel, and polynomial kernel (degree = 3), with the penalty parameter C set to values in the range 0.1, 10, 100. Utilizing a dataset consisting of 2000 normal trajectories and 200 anomalous trajectories (false alarms), this study computed the F1-score through five-fold cross-validation.

**Figure 16:**
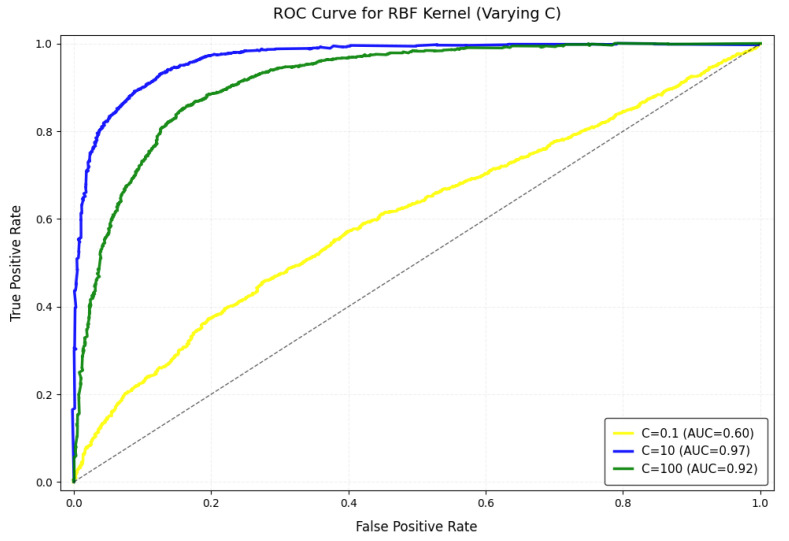
ROC curve for RBF Keynel. (The dotted line in the figure represents the performance benchmark for the random guess of the classifier, which has an AUC value of 0.5).

According to Figure 16, it can be known that:

Kernel Function Comparison:

- RBF Kernel (γ=0.1): AUC = 0.94, optimal performance.
- Sigmoid Kernel (γ=0.1): AUC = 0.82, second-best performance.
- Linear Kernel: AUC = 0.72, suitable for simple scenarios.
- Polynomial Kernel (degree = 3): AUC = 0.79, no obvious advantage.

**Figure 17:**
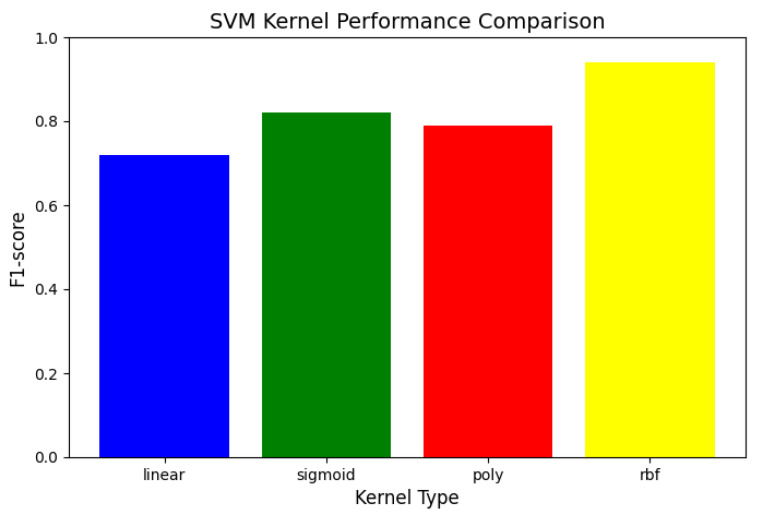
SVM Kernel performance comparison.

According to Figure 17, it can be known that:

Penalty Parameter C:

- C = 0.1: F1-score = 0.6, poor classification performance.
- C = 10: F1-score = 0.97, optimal performance.
- C = 100: F1-score = 0.92, performance degradation due to overfitting.

Overall, the Q-IMM-MHT algorithm exhibits exceptional performance in both global association accuracy and average association accuracy, while also maintaining lower rates of association errors and missed associations. This significantly enhances the overall effectiveness of trajectory association. Its capacity to sustain high association performance amidst target maneuvering and prolonged interruptions highlights its excellent adaptability to varying environments and promising application prospects.

## 4. Discussion

This paper introduces an enhanced algorithmic framework named Q-IMM-MHT, which integrates MHT with IMM to address the challenges of real-time multi-target tracking in the presence of strong clutter interference and trajectory interruptions. By incorporating a Q-learning-based adaptive model-switching strategy, as well as mechanisms for anomaly detection and trajectory recovery, and dynamic multi-hypothesis management, Q-IMM-MHT significantly improves the robustness and stability of the tracking system. Experimental results demonstrate that, in complex scenarios, Q-IMM-MHT outperforms traditional methods, particularly in maintaining trajectory continuity while substantially reducing both misassociation rates and false association rates in environments characterized by target trajectory interruptions and high false alarm rates.

The primary contributions of this work include the optimization of the model switching strategy through reinforcement learning and the integration of SVM techniques for anomaly detection and trajectory recovery. These advancements enhance the adaptability and predictive accuracy of multi-target tracking systems in complex environments. Compared to existing methods, Q-IMM-MHT exhibits superior robustness and higher prediction accuracy under highly dynamic conditions, multiple target interferences, and prolonged interruption periods. Future research can focus on further optimizing the computational efficiency of the algorithm, particularly in multi-target scenarios and more complex motion patterns. Exploring more efficient hypothesis management and trajectory update methods will enhance the algorithm’s real-time performance and scalability. Additionally, integrating advanced technologies such as deep learning with Q-IMM-MHT could further improve its adaptability and predictive precision in complex environments.

## Figures and Tables

**Table 1:** Association evaluation metrics for different algorithms across targets.

Interrupt Frames	EKF algorithm					IMM algorithm				
	Tar1	Tar2	Tar3	Tar4	Tar5	Tar1	Tar2	Tar3	Tar4	Tar5
6	122	91	75	83	66	388	246	217	226	185
12	16	11	0	3	0	195	168	153	172	133
18	0	0	0	0	0	82	66	51	59	42
24	0	0	0	0	0	6	3	0	0	0
30	0	0	0	0	0	0	0	0	0	0
Interrupt Frames	CIMM algorithm					Q-IMM-MHT algorithm				
	Tar1	Tar2	Tar3	Tar4	Tar5	Tar1	Tar2	Tar3	Tar4	Tar5
6	392	251	220	229	191	500	500	500	500	500
12	202	175	154	176	136	500	500	500	500	500
18	86	71	53	57	45	485	468	423	422	377
24	7	4	1	0	0	362	331	314	323	297
30	0	0	0	0	0	283	261	248	255	231

**Table 2:** Different algorithms correlate evaluation parameters.

Frame	EKF algorithm				IMM algorithm			
	$R_{ga}$	$R_{ta}$	$R_{fa}$	$R_{na}$	$R_{ga}$	$R_{ta}$	$R_{fa}$	$R_{na}$
6	0	0.1748	0.4468	0.3784	0.061	0.5048	0.3088	0.1864
12	0	0.012	0.6434	0.3446	0.016	0.3284	0.4144	0.2572
18	0	0	0.5876	0.4124	0	0.12	0.5788	0.3012
24	0	0	0.5338	0.4662	0	0.0036	0.6428	0.3536
30	0	0	0.4258	0.5742	0	0	0.5734	0.4266
Frame	CIMM algorithm				Q-IMM-MHT algorithm			
	$R_{ga}$	$R_{ta}$	$R_{fa}$	$R_{na}$	$R_{ga}$	$R_{ta}$	$R_{fa}$	$R_{na}$
6	0.064	0.5132	0.323	0.1638	1	1	0	0
12	0.018	0.3372	0.4202	0.2426	1	1	0	0
18	0.004	0.1248	0.5628	0.3124	0.592	0.87	0.122	0.008
24	0	0.0048	0.6436	0.3516	0.514	0.6508	0.3382	0.011
30	0	0	0.6128	0.3872	0.463	0.5112	0.4748	0.014

**Table 3:** Comparative performance analysis table before and after SVM optimisation.

Norm	Pre-Optimization	SVM Optimization	Enhancement
Single-frame processing time	−0.11–0.05 s	0.02–0.09 s	118–280% increase
real-time margin	0.3–0.36 s	0.16–0.23 s	34–47% increase
Number of Supportable Targets	3–5 targets	5–8 targets	60% increase

## Data Availability

The raw data supporting the conclusions of this article will be made available by the first authors upon request.

## Abbreviations

The following abbreviations are used in this manuscript:

UKF	Unscented Kalman Filter
IMM	Intel Mobile Module
CIMM	Centralized Interacted Multiple Model
SVM	Support Vector Machine
TPM	Transition Probability Matrix
